# Cabergoline-induced cerebrospinal fluid fistulae in macroprolactinomas

**DOI:** 10.1007/s00423-025-03722-8

**Published:** 2025-05-06

**Authors:** Elvis J. Hermann, Sabine Hertz, Makoto Nakamura, Christoph Terkamp, Thomas M. Kinfe, Stefan Stolle, Holger Leitolf, Rudolf Fahlbusch, Joachim K. Krauss

**Affiliations:** 1 Department of Neurosurgery, Hannover Medical School, Carl-Neuberg-Str.1, 30625 Hannover, Germany; 2 Department of Neurosurgery, Klinikum Köln-Merheim, Köln, Germany; 3 Department of Gastroenterology, Hepatology and Endocrinology, Hannover Medical School, Hannover, Germany; 4 Department of Neurosurgery, Mannheim Center for Neuromodulation and Neuroprosthetics, Medical Faculty Mannheim, Ruprecht-Karl-University, Heidelberg, Germany; 5 Department of Laryngology, Rhinology and Otology, Hannover Medical School, Hannover, Germany; 6 Department of Neurosurgery, International Neuroscience Institute, Hannover, Germany

**Keywords:** Cerebrospinal fluid leak, Cerebrospinal fluid fistula, Rhinorrhea, Dopamine agonist, Prolactinoma, Pituitary adenoma

## Abstract

**Purpose:**

Cerebrospinal fluid (CSF) rhinorrhea is a rare complication after dopamine agonist treatment of macroprolactinomas. Up to 90% need surgical repair, however, there are controversial opinions on the necessity of tumor resection upon this occasion. Here we present our long-term follow-up experience in patients who underwent surgical repair of the CSF leak or observation.

**Methods:**

We report a series of three patients who presented with cabergoline-induced CSF rhinorrhea with long-term follow-up up to 170 months. Two patients underwent endoscopic transnasal-transsphenoidal surgical repair of CSF rhinorrhea by fat graft and fibrin glue without tumor removal. In another patient, CSF rhinorrhea resolved spontaneously after two weeks without recurrence.

**Results:**

All three patients had no recurrence of CSF rhinorrhea during long-term follow-up up to 170 months. One patient with surgical CSF leak repair was asymptomatic with continued medication at long-term follow-up of 116 months. Tumor progression occurred 21 months after CSF leakage repair in another patient after cessation of dopamine agonist treatment and necessitated tumor debulking. The patient with continued medication without surgery had no recurrence of CSF rhinorrhea on long-term follow-up of 170 months.

**Conclusion:**

The optimal management of CSF fistulae due to tumor shrinkage of macroprolactinomas after dopamine agonist therapy remains to be defined. Exceptionally, medication-induced CSF fistulae in response to tumor shrinkage may close spontaneously without recurrence. If persistent, transnasal-transsphenoidal closure of CSF fistulae represents an efficient treatment and dopamine agonist treatment may be continued. Thus, we recommend early surgical repair.

## Introduction

Medical treatment has replaced surgical resection of prolactinomas over the past four decades after introduction of bromocriptine in 1971 and of cabergoline in 1986 [1, 2], although more recently the published advantages of surgery have been reconsidered [3–7]. The selective dopamine D2 receptor agonist cabergoline is now recommended for primary treatment according to the evidence-based Endocrine Society Clinical Practice Guideline since it has been shown to be superior in terms of response rate, resistance, tolerance and therapy regimen [8, 9]. Both, clinical improvement and tumor shrinkage are observed in the majority of patients on long-term follow-up [9–12].

Cerebrospinal fluid (CSF) rhinorrhea has been thought to be unusual after medical treatment of prolactinomas, but it has been reported occasionally in the form of case reports or small case series [13–27]. Since, it was first described in the early 1980s, various treatment algorithms have been suggested. There are several open questions to be solved including the issue whether or not the tumor should be resected at the time of CSF fistula repair [13, 18, 22, 28]. A major problem to evaluate the different therapies which have been proposed is the limited length of follow-up in most instances.

Here, we report our long-term follow-up experience in three patients with cabergoline-induced CSF rhinorrhea who underwent either surgical repair of the CSF leak or observation.

## Materials and methods

The database of patients with pituitary adenomas presenting at the Department of Neurosurgery at Hannover Medical School between 2005 and 2023 was screened retrospectively. Three patients with macroprolactinomas and medication-induced CSF rhinorrhea were identified. The medical records and imaging studies were reviewed and follow-up was supplemented. Data collected included patient demographics, symptoms at the time of diagnosis of prolactinoma, tumor expansion, serum prolactin before initiation of cabergoline therapy, weekly cabergoline dose administered, time span between initiation of cabergoline therapy and occurrence of CSF rhinorrhea, method of CSF leak repair, and histopathology.

Serum prolactin was measured excluding the “high dose hook effect” by dilution of 1:10 and 1:100. Recent follow-up was obtained in all patients.

## Results

There were two men, and one woman. Age ranged between 19 and 66 years at the time of the occurrence of the CSF fistula (see Table 1). There was no incidence of meningitis. The time span between initiation of cabergoline therapy and occurrence of rhinorrhea was short (4–6 weeks in all cases). In one patient (case 2), CSF rhinorrhea resolved spontaneously. The other two patients underwent exploration via an endoscopic transnasal-transsphenoidal procedure and successful sealing of the dural defect with autologous fat graft and fibrin glue was achieved. For visualization of the CSF leak, intrathecal injection of fluorescein (5% fluorescein diluted 1:10, 0.01 ml per kg body weight) was performed on the evening before surgery. Both patients with surgical repair were treated with a lumbar drain for 7 and 12 days, respectively.

**Table 1:** Demographic and clinical characteristics of patients with macroprolactinomas and cabergoline-induced cerebrospinal fluid fistulae

Patients					Tumor expansion prior to cabergoline treatment							CSF leak appearance		CSF rhinorrhea treatment and outcome		
Case #	Sex	Age	Initial PRL ng/ml	Symptoms on initial diagnosis	Size axial x CC mm	Optic chiasm	Carotid encase- ment	Cavernous sinus invasion	Sphenoid sinus invasion	Posterior ethmoid cells invasion	Clivus invasion	CAB dose/ week	Weeks to CSF leak	Surgical repair	LD post-op days	Site of CSF leak
1	F	19	n/a	Galactorrhea, secondary oligo-menorrhea	77 × 37	Slightly compressed	L + R	L + R	Completely filled	L + R	yes	2x 0,25 mg	6	yes	12	clivus
2	M	34	31,900	Nausea	80 × 36	Slightly compressed	L + R	L + R	Filled	L + R	yes	7x 0,5 mg	4	no (spontaneous closure after 3 weeks)	-	sella
3	M	66	3622	Left eye: upper temporal quadrant anopia	31 × 35	Compressed	L + R	L + R	L + R Small part	no	yes	7x 0,25 mg	6	yes	7	sella

PRL = prolactin, CC = craniocaudal, CAB = cabergoline, L = left, R = right, LD = lumbar drain

CSF rhinorrhea did not recur in any instance. The follow-up period ranged between 116 and 170 months. One patient underwent partial tumor resection in another clinic due to tumor growth after cessation of medication 21 months following CSF fistula repair.

### Case vignettes

#### Case 1

A 19-year-old woman was admitted with increasing headaches. At age 15 a prolactinoma had been diagnosed at another hospital. Cabergoline therapy was installed and the tumor responded well. Galactorrhea and secondary oligomenorrhea resolved, serum prolactin had dropped to normal levels.

(6.3 ng/ml). When she had become pregnant cabergoline was exchanged for bromocriptine which she took only sporadically due to nausea.

**Fig. 1:**
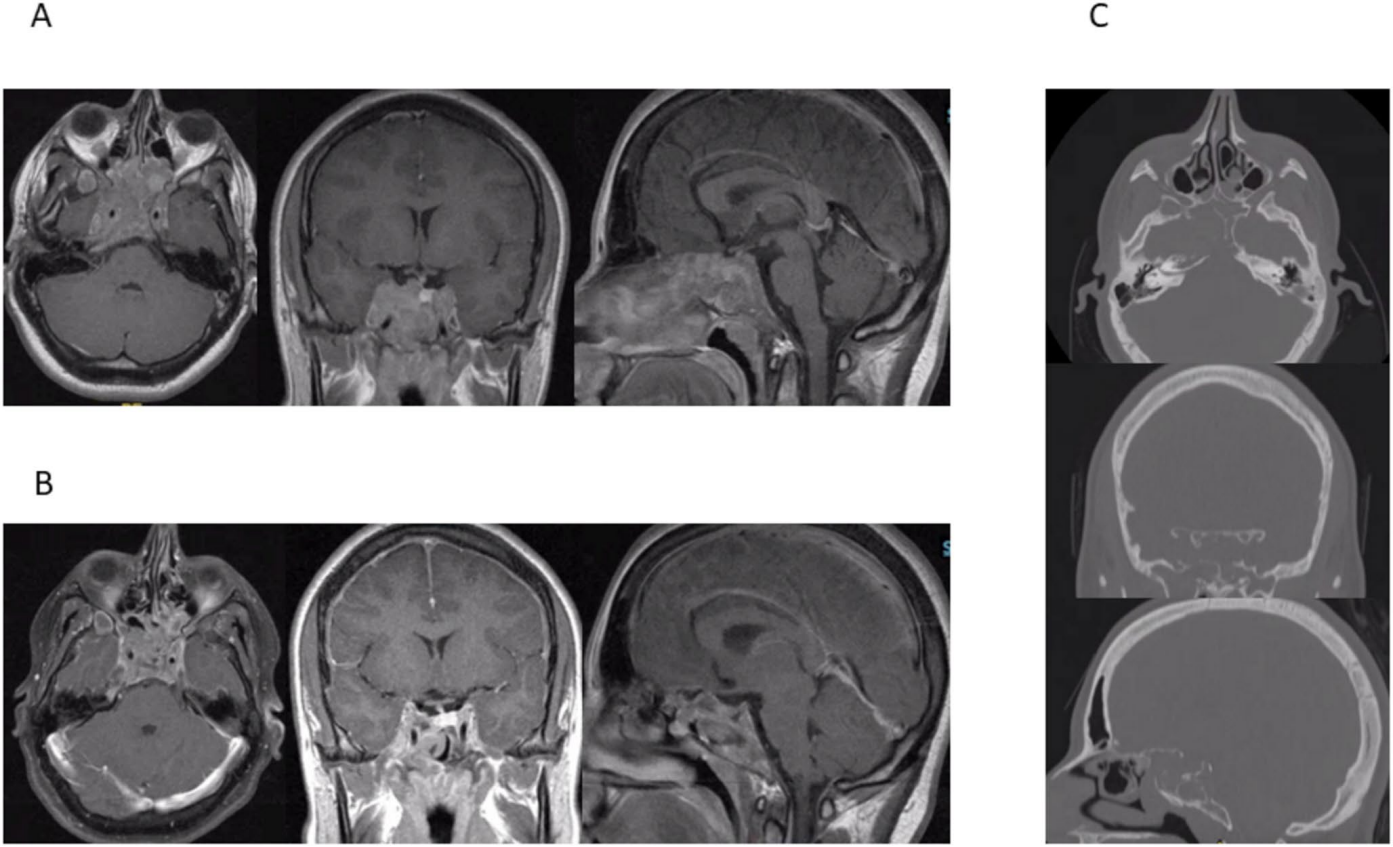
Imaging studies of patient 1 (19-year-old woman). **A** T1-weighted contrast enhanced MRI studies before re-administration of cabergoline therapy. The extensive pituitary tumor is widely invading the skull base, expanding to the right petrous bone, the cavernous sinus, the right sphenoid sinus and the clivus. **B** T1-weighted contrast enhanced MRI studies two months after re-administration of cabergoline therapy. The tumor has shrinked substantially. A small intratumoral cavity filled with air is visible. **C** CT imaging in bone window show the bony defects in the skull base (sella, clivus and sphenoid sinus wall)

Upon admission to our service at age 19 ophthalmologic examination did not reveal visual field defects. Endocrinological analysis under bromocriptine therapy showed suppressed but moderately increased serum prolactin (187 ng/ml), raised serum IGF1 (1118 ng/ml) and elevated serum HGH (46 ng/ml) levels. She also had stigmata of acromegaly. MRI now showed a giant pituitary adenoma (Fig. 1) with a maximum axial diameter of 77 mm expanding into the nasal cavity. Computed tomography (CT) imaging of the skull base showed extensive bony defects of the sella, the clivus and the sphenoid sinus wall due to compression and erosion. The tumor had invaded the posterior ethmoid cells. A biopsy was obtained via a transnasal-transsphenoidal approach. Histopathological examination confirmed the initial diagnosis of prolactinoma. No GH secreting cells were found in the tumor biopsy. The cabergoline regimen was re-established with doses of 0.25 mg biweekly. She again had a good response to cabergoline with a serum prolactin level of 40 ng/ml, but she still had elevated serum levels for GH (207.10 ng/ml) and for IGF-1 (731 ng/ml). She refused to take additional medication.

She was re-admitted 10 weeks later with CSF rhinorrhea which had become apparent 4 weeks earlier. MRI imaging showed a significant reduction in tumor volume (Fig. 1). A CT scan demonstrated bony defects of the skull base. A transnasal-transsphenoidal exploration was performed by endoscopic technique. On the evening prior to the procedure, fluorescein was administered intrathecally. Intraoperatively a defect was detected in the transition zone to the upper clivus which was repaired with a fat plug and fibrin glue. A lumbar drain was installed for twelve days. There was no recurrence of CSF leakage thereafter and medication with cabergoline was continued (0.25 mg three times weekly). Serum prolactin at 11 months postoperatively was 40ng/ml. After medication was stopped during another pregnancy massive tumor growth occurred with severe headaches and oculomotor disturbances. The tumor then was debulked via a transnasal approach (21 months after CSF fistula repair) followed by postoperative radiotherapy. Further follow-up with continued medication was unremarkable without recurrence of the CSF fistula.

#### Case 2

**Fig. 2:**
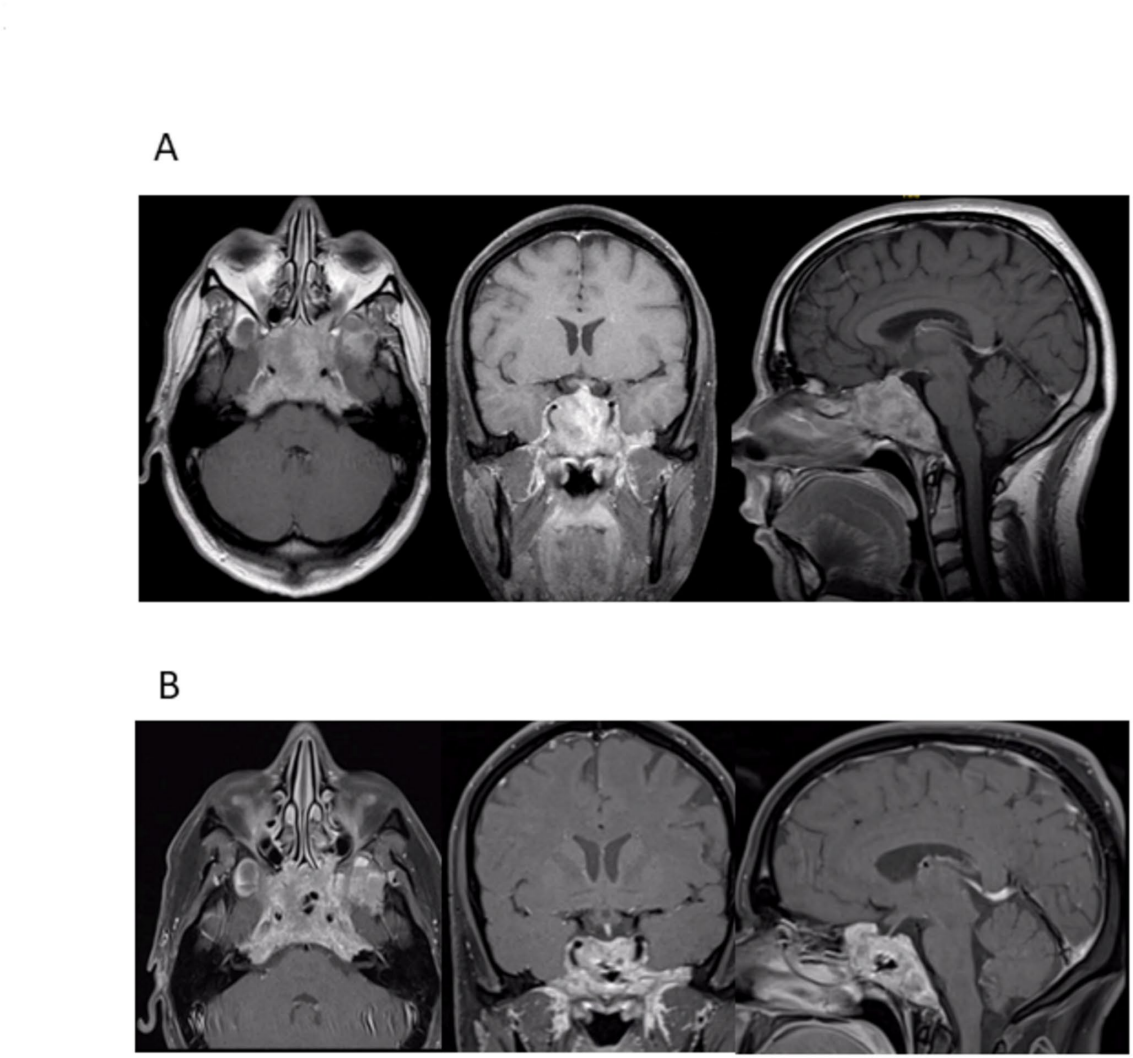
Imaging studies of patient 2 (34-year-old man). **A** T1-weighted contrast enhanced MRI studies show a giant prolactinoma with a maximum diameter of 8 cm encasing both ICAs and invading the cavernous sinus and the clivus. **B** T1-weighted contrast enhanced MRI studies 10 days after initiation of cabergoline therapy. There is massive tumor shrinkage with a small depot of air in the center of the tumor

A 34-year-old man presented with nausea. MRI showed an osteolytic lesion of the skull base and a giant prolactinoma expanding from the foramen magnum to the sella and the sphenoid sinus, and CT imaging demonstrated bony defects in the anterior cranial fossa (Fig. 2). Endocrinological work-up revealed a serum prolactin level of 31.900 ng/ml. Medication with cabergoline 0.25 mg daily was initiated. Serum prolactin dropped to 690 ng/ml within days. Due to a slight increase in serum prolactin some days later, the daily cabergoline dosage was subsequently increased to 0.5 mg. The serum prolactin then dropped to 308 ng/ml. MRI 10 days after initiation of cabergoline therapy showed a substantial reduction in tumor volume (Fig. 2).

The patient subsequently noted recurrent epistaxis. Two weeks thereafter rhinorrhea became evident. The nasal discharge was tested for beta-transferrin which was significantly raised (35 mg/dl). Surgical repair of the fistula was suggested, but the patient opted for a wait-and-see strategy. Medication was continued, and the patient was monitored with close surveillance. CSF discharge resolved spontaneously three weeks later and did not recur during follow-up up to 170 months. The serum prolactin at the last follow-up was 43.1 ng/ml.

#### Case 3

**Fig. 3:**
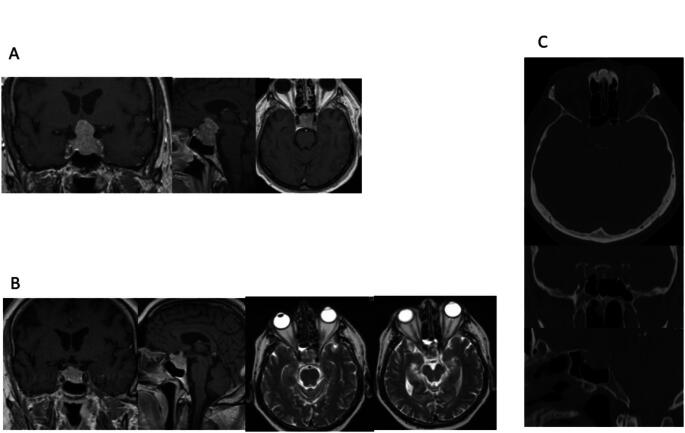
Imaging studies of patient 3 (66-year-old man). **A** T1-weighted contrast enhanced MRI studies demonstrate a macroadenoma with para- and suprasellar expansion through the sellar diaphragm. **B** T1-weighted contrast enhanced MRI studies and T2-weighted MRI studies 6 weeks after cabergoline therapy show considerable shrinkage of the tumor. Note the fluid level in the posterior ethmoid cells on the right side. **C** CT imaging in bone window show the bony destruction of the upper clivus with tumor invasion into the ethmoid sinus and the nasal cavity

A 66-year-old man presented with a visual field defect. MRI showed an invasive pituitary macroadenoma expanding to the parasellar, suprasellar and infrasellar regions. Ophthalmologic examination demonstrated a temporal upper quadrant anopia for the left eye. MRI showed a tumor expanding through the sellar diaphragm in a waist-like formation considerably compressing the optic chiasm, and CT demonstrated invasion of the superior sphenoid sinus and erosion of the sellar walls (Fig. 3). Endocrinological work-up revealed a serum prolactin level of 3622 ng/ml.

Medication with cabergoline at a dosage of 0.25 mg every second day was initiated. The serum prolactin level 10 days later showed a decrease to 2449 ng/ml. The cabergoline regime was.

subsequently changed to 0.25 mg daily. MRI showed a significant decrease in tumor volume. Six weeks later the patient presented with rhinorrhea. MRI now showed fluid in the posterior ethmoid cells on the right side (Fig. 3). Endoscopic transnasal exploration was performed seven weeks thereafter. On the evening prior to surgery, the patient received fluorescein intrathecally. Intraoperatively a defect could be detected at the anterior sellar wall where the tumor had invaded the skull base. The defect was repaired with an autologous fat plug, a fibrin coated sponge and a bone graft obtained from the perpendicular lamina. A lumbar drain was inserted for seven days. Cabergoline was tapered down to a maintenance dose of 0.25 mg weekly. There was no recurrence of CSF leakage during follow-up up to 116 months. The serum prolactin at the last follow-up was 0.8 ng/ml.

## Discussion

Dopamine agonists effectively inhibit prolactin secretion in 90% of patients with prolactinoma and lead to substantial tumor shrinkage due to reduction of the lactotrophs’ cell size [10, 29]. Accordingly, in the three patients described here the macroprolactinoma quickly responded to cabergoline treatment with decreased serum prolactin levels and significant tumor shrinkage.

Although histologically benign, the growth pattern of prolactinomas often is invasive with bony destructions. In a study of 115 patients with pituitary macroadenomas where tumor invasiveness was assessed on the basis of MRI, prolactinomas were found to be more frequently invasive (67%) as compared to GH-secreting (46%) and nonsecreting (16%) pituitary adenomas [30]. In a series of surgical specimens of 60 patients with pituitary adenomas, microscopic dural infiltration was found in 91% of cases and in all patients (8 cases) with macroprolactinomas [31]. Prolactinomas are known to expand preferentially into the infrasellar space and to invade the sphenoid bone, occasionally reaching out to the nasopharynx or nasal cavity [32]. This growth pattern may be explained by topographic aspects as the lactotrophs are mainly located in the caudal part of the pituitary gland. Similarly, GH-secreting adenomas expand preferentially into the infrasellar space [33]. Prolactinomas are also known to invade diffusely the cavernous sinus and the clivus or skull base [30].

Effective treatment of invasive giant prolactinoma with dopamine agonists resulting in rapid tumor shrinkage may expose the eroded dura and skull base formerly plugged by adenoma masses [34]. In accordance with this, our three patients had substantial infrasellar invasion, and CSF leakage occurred shortly (4 to 6 weeks) after the initiation of cabergoline treatment.

The true incidence of dopamine agonist therapy-induced CSF leakage in macroprolactinomas has not been fully elucidated. While it is not mentioned in large follow-up studies on prolactinoma treatment and it is reported usually only in the form of case reports, studies concentrating on the subject in small series indicate that it could occur in up to 8.7–13.3% of patients [18, 24, 28]. Discontinuation of dopamine agonist treatment has been suggested as an option for conservative management of CSF fistulae in macroprolactinoma [17]. This approach, however, might not be feasible in symptomatic patients. Therefore, a wait-and-see strategy with continued medication and close surveillance might be another option in patients unwilling to undergo surgery as exemplified in the case of patient 2 in our report.

Cabergoline-induced CSF leakage represents a potential side-effect of an excellent dopamine agonist response with major reduction in tumor size and serum prolactin level. Risk factors for the occurrence of CSF fistulae in macroprolactinomas include higher initial prolactin levels, and invasion of the cavernous sinus and the sphenoid sinus [26, 27].

Several options for surgical management of CSF leakage secondary to medication-induced shrinkage of macroprolactinomas have been suggested [28]. Transnasal-transsphenoidal approaches are being preferred, and endoscopic techniques have been used more frequently [14, 18, 35, 36]. The CSF leaks may be closed by fat grafts, fascia lata grafts or periostal nasoseptal flaps. Postoperative lumbar drainage has been commonly recommended, because reccurence after primary CSF leakage may thus be prevented [14, 37, 38].

There is little consensus on the management of medication-induced CSF leaks in prolactinomas. Nevertheless, there is no agreement about the timing of readministration of dopamine-agonist therapy and CSF rhinorrhea can reoccur making surgical revision necessary [39]. Regarding our two surgically managed cases, we may confirm the utility of a perioperative lumbar drain, however, a wait-and-see strategy with continued medication, as observed in one of our cases, may also lead to cessation of the CSF leak.

There is disagreement whether (partial) tumor resection should be performed upon the occasion of repair of the CSF leak [17, 28, 40]. In a recent publication three cases with medication-induced CSF leakage were reported, in which transnasal-transsphenoidal closure was performed. Partial tumor resection was performed to visualize the defect in the dura and the skull base to better achieve reconstruction and endoscopic closure [41]. It has been stated that tumor debulking in macroprolactinoma, in general, should be preserved for patients with dopamine agonist resistance characterized by inactive or absent D2-receptors [42]. Partial tumor resection at the time of CSF repair might have a negative effect since further tumor shrinkage is expected with continued medical treatment.

Carbogoline has replaced bromocriptine for treatment of prolactinomas [11, 16–18, 20, 22–24]. Interestingly, in comparison when bromocriptine was used for treatment CSF fistulae seemed to occur later [13, 15, 20, 43].

CSF leakage following prolactinoma size reduction has been described as the “uncorking effect” of dopamine agonist therapy unplugging an eroded part of the skull base [43]. With regard to the risk of meningitis it has been recommended to repair dural defects urgently. Interestingly, a case of a patient with frequent periods of CSF rhinorrhea refusing surgical repair has been reported without occurrence of infectious complications during a follow-up period of 4 years [22]. There is no agreement for the timing of repair to avoid the risk of life-threatening meningitis. However, the impact of meningitis on healing CSF leaks is a matter of debate. It was discussed that the inflammation may cause adhesions contributing to the cessation of the CSF rhinorrhea following dopamine-agonist therapy [44]. Nevertheless, CSF leakage repair is necessary in the vast majority of cases [2, 17]. According to a review by Lam and colleagues nonsurgical management of CSF leaks in patients with macroprolactinomas was successful in only 4/52 patients while 46/52 underwent surgical intervention [28]. Patients with medical treatment of macroprolactinomas should be informed about the potential hazardous complications of CSF rhinorrhea and should be monitored closely for CSF leakage.

There are several limitations of our study. First of all, the small sample size limits its generalizability. Second, the retrospective design may result in biased case-based conclusions.

## Conclusions

Our study underpins the necessity to carefully monitor patients with macroprolactinomas for CSF rhinorrhea and its complications closely after medical treatment was started. Although dopamine agonist-induced CSF rhinorrhea might resolve spontaneously in exceptional cases, we recommend early surgical repair when a CSF leak has clearly been demonstrated by an endoscopic transnasal-transsphenoidal approach. Further studies on the usefulness of tumor removal at the time of CSF leak repair are necessary.

## Data Availability

No datasets were generated or analysed during the current study.
